# Secular Trends in the Incidence of and Mortality Due to Alzheimer’s Disease and Other Forms of Dementia in China From 1990 to 2019: An Age-Period-Cohort Study and Joinpoint Analysis

**DOI:** 10.3389/fnagi.2021.709156

**Published:** 2021-09-03

**Authors:** Yongliang Gao, Xiaonan Liu

**Affiliations:** ^1^Department of Neurology, The Fourth Affiliated Hospital of China Medical University, Shenyang, China; ^2^Department of Neurological Function Examination, The Fourth Affiliated Hospital of China Medical University, Shenyang, China

**Keywords:** Alzheimer’s disease, dementia, China, epidemiology, incidence

## Abstract

**Background:**

The number of individuals with dementia is increasing, which negatively affects families, communities, and health care systems worldwide. The changes in the incidence of and mortality due to Alzheimer’s disease and other forms of dementia at the national level in China have remained unknown over the past three decades.

**Methods:**

Following the general analytical strategy used in the Global Burden of Disease Study (GBD) 2019, the age- and sex-specific incidence and mortality rates for dementia in China were analyzed. Trends in the incidence of and mortality due to dementia from 1990 to 2019 were assessed by joinpoint regression analysis. The effects of age, period and cohort on the incidence of and mortality due to dementia were estimated by an age-period-cohort model.

**Results:**

The age-standardized incidence and mortality rates per 100,000 population were 103.83 (95% UI, 87.93–118.87) and 23.32 (95% UI, 5.66–61.31), respectively, for dementia in 2019. From 1990 to 2019, a significant average annual percentage change (AAPC) in the age-standardized incidence rate was observed in both males [0.49% (95% CI, 0.43–0.55%)] and females [0.31% (95% CI, 0.24–0.38%)], and the age-standardized mortality rate significantly increased in males [0.42% (95% CI, 0.31–0.53%)]. The population aged 55–59 years had the highest AAPC in the incidence of dementia [0.87% (95% CI, 0.81–0.93%)]. The age effect showed that the relative risks (RRs) of incident dementia and dementia-associated mortality increased with age among males and females, and individuals aged 60 years and older had significantly higher RRs. The RR of incident dementia increased with time, and the RR started to substantially increase in 2009. The cohort effect showed that the incidence decreased in successive birth cohorts.

**Conclusion:**

Alzheimer’s disease and other forms of dementia continue to become more common among males and females in China, and the associated mortality rate in males significantly increased from 1990 to 2019. Early interventions should be implemented to reduce the burden of dementia on individuals at high risk in China.

## Introduction

With the aging of the global population, Alzheimer’s disease and other forms of dementia have become major and increasingly severe global public health threats (GBD 2016 Disease and Injury Incidence and Prevalence Collaborators, 2017). In 2016, the global numbers of prevalent cases and deaths due to dementia were 43.8 million and 2.4 million, respectively (GBD 2016 Disease and Injury Incidence and Prevalence Collaborators, 2017). Notably, dementia is the fifth leading cause of death worldwide (GBD 2016 Disease and Injury Incidence and Prevalence Collaborators, 2017). The World Alzheimer Report 2019 estimated that the global prevalence will increase to 152 million by 2050 (Alzheimer’s Disease International, 2019). Dementia is a more serious public health problem in low- and middle-income countries (GBD 2016 Disease and Injury Incidence and Prevalence Collaborators, 2017). According to the classification system produced by the World Bank in 2015, 58% of people with dementia live in low- and middle-income countries, and this proportion is expected to increase to 63% by 2030 and to 68% by 2050 (Prince et al., 2015). The burden imposed by dementia is economically devastating because individuals with dementia are generally disabled and dependent. Currently, the annual cost associated with dementia is estimated to be US$1 trillion, and this number will double by 2030 (Alzheimer’s Disease International, 2019).

In China, due to the prolonged life expectancy and the growth of the elderly population, dementia has become an important public health problem. The 2015 World Alzheimer’s Disease Report estimated that there are more than 9.5 million Alzheimer’s disease patients in China, accounting for 20% of the world’s total number of Alzheimer’s disease patients (Prince et al., 2015). By 2050, the number of people with dementia in China is expected to increase to more than 35 million (Wang M. et al., 2019). Several previous cross-sectional studies estimated that the prevalence of Alzheimer’s disease and other forms of dementia is approximately 5–6% in the Chinese population (Zhang et al., 2005; Ding et al., 2015). A systematic review estimated that the prevalence of dementia increased from 1.8 to 2.6% in the 65–69-year-old population and from 42.1 to 60.5% in the 95–99-year-old population (Chan et al., 2013). Based on data from the Chinese Longitudinal Healthy Longevity Survey, it was estimated that the incidence of cognitive impairment among older Chinese people decreased from 1998 to 2014 (Gao et al., 2017). Furthermore, a previous study showed increasing trends in dementia prevalence and incidence in elderly Chinese individuals during the period from 1987 to 2016 (Ding et al., 2020). In general, these surveys on the incidence of and mortality due to Alzheimer’s disease and other forms of dementia were mainly conducted among specific subpopulations and in earlier years, and the disease incidence and mortality rates might currently be higher as a result of the aging of the Chinese population (Gao et al., 2017; Ding et al., 2020).

Given the limited epidemiological information about Alzheimer’s disease and other forms of dementia in the Chinese population, more updated estimations are needed to help guide future research on disease control and prevention strategies. Age-period-cohort analysis has become a popular method of assessing the impacts of chronological age, time period, and birth cohort on outcomes, such as disease incidence and mortality rates (Clayton and Schifflers, 1987). The age effect represents the different risks of outcomes in various age groups (Clayton and Schifflers, 1987). The period effect reflects the influence of a series of complex historical events and environmental factors (Clayton and Schifflers, 1987). The birth cohort effect reflects the characteristics of each generation and considers the risk factors and exposure to environmental factors present in early life (Clayton and Schifflers, 1987). The results of age-period-cohort analyses may provide epidemiologists with important clues to or help them generate hypotheses about the etiologies of diseases. This study involved the analysis of data from the Global Burden of Disease (GBD) 2019 survey to examine the temporal trends in the incidence of and mortality due to Alzheimer’s disease and other forms of dementia in China and to explore the net age, period, and cohort effects with the age-period-cohort framework.

## Materials and Methods

### Study Population and Data Collection

The GBD 2019 study used all available up-to-date sources of epidemiological data and improved standardized methods to provide a comprehensive assessment of health loss due to 369 types of diseases and injuries and 87 risk factors in 204 countries and territories (GBD 2019 Diseases and Injuries Collaborators, 2020; GBD 2019 Risk Factors Collaborators, 2020). Details of the methodology used in the GBD 2019 study have been explained in previous studies and are presented in Supplementary Material (GBD 2019 Diseases and Injuries Collaborators, 2020; GBD 2019 Risk Factors Collaborators, 2020). The data analysis was completed on April 15, 2021. The Institutional Review Board of the Fourth Affiliated Hospital of China Medical University determined that this study did not require approval because it used publicly available data.

### Statistical Analysis

The age-standardized rates and their average annual percentage changes (AAPCs) were calculated with linear regression analysis to assess the incidence of and mortality due to Alzheimer’s disease and other forms of dementia. All the rates are reported per 100,000 population. The 95% uncertainty interval (UI) for each quantity was calculated in our study. Significance in all the analyses was assessed at the 0.05 level, and all hypothesis tests were two-sided.

Joinpoint regression analysis was used to assess trends in the disease burden of Alzheimer’s disease and other forms of dementia. Joint Command Line Version 4.5.0.1 joinpoint software was provided by the United States National Cancer Institute Surveillance Research Program. This software tracks trends in data over time and then fits the simplest model possible to the data by connecting several different line segments on a logarithmic scale. These segments are known as “joinpoints,” with the simplest model (i.e., 0 joinpoints) being a straight line. As more joinpoints are added, each is tested for significance using a Monte Carlo permutation method. The AAPCs were calculated to assess the trends, and the *Z*-test was used to assess whether the AAPCs were significantly different from zero. When describing trends, the terms increase or decrease are used when the slope of the trend was found to be statistically significant.

To assess the relative risks (RRs) in the population in a particular year and the accumulation of health risks since birth, we used the age-period-cohort model. This model allows the analysis of the independent effects of age, period, and cohort on temporal trends in the incidence of and mortality due to Alzheimer’s disease and other forms of dementia. The age-period-cohort model provides a useful parametric framework that complements standard non-parametric descriptive methods. In this model, the collected data were stratified into successive 5-year age groups and consecutive 5-year periods. The incidence and mortality rates for dementia were recorded in successive 5-year age groups (from 40–44 to 75–79 years), consecutive 5-year periods (from 1994 to 2019), and corresponding consecutive 5-year birth cohorts from 1915–1919 to 1975–1979. The age–period–cohort analysis with the intrinsic estimator method provided estimations of the coefficients for the age, period, and cohort effects. These coefficients were transformed into exponential values [exp(coef.) = e^*coef.*^], which denotes the RRs of incident dementia and dementia-associated mortality for a particular age, period, or birth cohort relative to the average level of all ages, periods, or birth cohorts combined. Age–period–cohort analysis was performed using STATA 15.0 software (StataCorp, College Station, TX, United States).

## Results

### Descriptive Analysis

**FIGURE 1:**
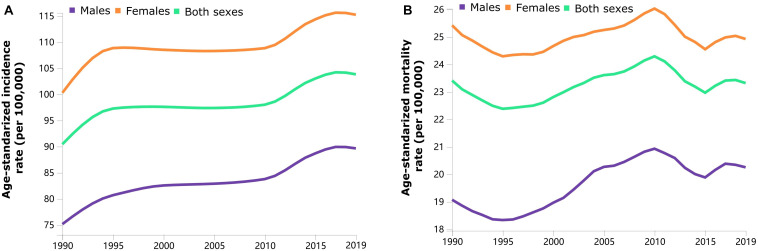
Trends in the sex-specific age-standardized incidence **(A)** and mortality **(B)** rates for Alzheimer’s disease and other forms of dementia in China from 1990 to 2019.

Trends in the sex-specific age-standardized incidence and mortality rates for dementia in China from 1990 to 2019 are shown in Figure 1. The sex-specific, age-standardized incidence and mortality rates for dementia fluctuated by calendar year. Generally, the age-standardized incidence rate has increased over the three decades. The sex-specific incidence and mortality rates for dementia stratified by age group in 1990 and 2019 are presented in Table 1. The age-standardized incidence [115.24 (95% UI, 97.62–132.73) vs. 89.65 (95% UI, 74.93–103.22) per 100,000 population] and mortality [24.92 (95% UI, 5.98–64.47) vs. 20.25 (95% UI, 4.80–57.38) per 100,000 population] rates were higher among females than among males in 2019. The age-standardized incidence and mortality rates changed from 90.44 (95% UI, 76.25–104.07) to 103.83 (95% UI, 87.93–118.87) and from 23.41 (95% UI, 5.40–64.04) to 23.32 (95% UI, 5.66–61.31) per 100,000 population in China from 1990 to 2019, respectively. The incidence and mortality rates increased with increasing age in both sexes.

**TABLE 1:** Sex- and age-specific rates of Alzheimer’s disease and other forms of dementia in China in 1990 and in 2019 and their average annual percentage changes (AAPC) from 1990 to 2019.

Categories	Males			Females			Overall		
	Rates in 1990 (95% UI)	Rates in 2019 (95% UI)	AAPC,% (95%CI)	Rates in 1990 (95% UI)	Rates in 2019 (95% UI)	AAPC,% (95%CI)	Rates in 1990 (95% UI)	Rates in 2019 (95% UI)	AAPC,% (95%CI)
Incidence									
40–44 years	4.4 (2.17–7.17)	4.47 (2.21–7.41)	0.15 (0.1–0.21)*	4.81 (2.41–7.88)	4.9 (2.44–8.04)	0.17 (0.13–0.22)*	4.6 (2.3–7.51)	4.68 (2.33–7.74)	0.17 (0.12–0.22)*
45–49 years	17.58 (11.13–25.19)	19.29 (12.1–27.99)	0.38 (0.34–0.42)*	19.64 (12.34–27.98)	21.47 (13.39–30.69)	0.35 (0.32–0.38)*	18.55 (11.73–26.53)	20.36 (12.79–29.2)	0.37 (0.34–0.4)*
50–54 years	35.18 (22.86–49.69)	42.29 (27.15–59.73)	0.65 (0.58–0.72)*	39.37 (25.41–55.6)	47.04 (30.09–66.58)	0.6 (0.53–0.67)*	37.15 (24.03–52.58)	44.65 (28.6–62.87)	0.64 (0.57–0.7)*
55–59 years	64.04 (43.27–90.34)	86.43 (57.57–119.97)	0.93 (0.87–0.99)*	73.08 (49.14–104.6)	94.84 (62.61–132.83)	0.8 (0.73–0.87)*	68.34 (46.28–96.57)	90.61 (60.17–126.33)	0.87 (0.81–0.93)*
60–64 years	106.8 (71.1–152.09)	148.13 (100.28–208.36)	0.88 (0.77–0.99)*	131.67 (87.24–189.54)	173.72 (115.92–248.25)	0.73 (0.63–0.82)*	118.86 (79.14–170.37)	160.87 (108.68–227.79)	0.81 (0.71–0.91)*
65–69 years	176.12 (116.84–262.57)	231.6 (153.89–342.54)	0.67 (0.56–0.79)*	238.62 (158.94–350.82)	324.07 (215.29–475.56)	0.68 (0.54–0.83)*	207.89 (138.05–307.74)	278.68 (186.29–409.52)	0.68 (0.55–0.81)*
70–74 years	345.55 (220.42–502.82)	440.18 (284.4–629.21)	0.62 (0.52–0.71)*	492.84 (317.19–709.63)	677.82 (451.69–960.68)	0.7 (0.56–0.85)*	424.54 (271.67–613.67)	561.98 (371.91–792.98)	0.63 (0.51–0.76)*
75–79 years	766.12 (522.34–1083.1)	954.76 (652.41–1346.14)	0.62 (0.54–0.69)*	1113.24 (760.84–1551.69)	1384.98 (956.7–1916.07)	0.47 (0.36–0.57)*	963.86 (656.88–1350.07)	1181.21 (813.66–1639.01)	0.45 (0.36–0.55)*
80–84 years	1384.36 (931.63–1918.8)	1630.32 (1107.75–2264.72)	0.51 (0.45–0.57)*	1952.14 (1325.67–2657.22)	2099.37 (1433.16–2922.43)	0.16 (0.1–0.21)*	1733.09 (1172.66–2374.03)	1893.35 (1287.45–2619.85)	0.22 (0.17–0.28)*
85–89 years	2108.97 (1467.06–2894.39)	2360.38 (1661.81–3252.72)	0.34 (0.27–0.41)*	2760.42 (1949.81–3779.14)	2735.93 (1913.04–3797.69)	−0.05 (–0.11–0.01)	2557.18 (1797.05–3500.72)	2604.45 (1825.1–3610.72)	0.04 (–0.02–0.11)
Age-standardized rate	75.14 (62.9–86.73)	89.65 (74.93–103.22)	0.49 (0.43–0.55)*	100.28 (84.67–115.63)	115.24 (97.62–132.73)	0.31 (0.24–0.38)*	90.44 (76.25–104.07)	103.83 (87.93–118.87)	0.33 (0.27–0.4)*
Mortality									
40–44 years	0.12 (0.01–0.43)	0.11 (0.01–0.37)	−0.29 (−0.42–0.15)*	0.12 (0.01–0.44)	0.1 (0.01–0.36)	−0.55 (−0.67–0.44)*	0.12 (0.01–0.43)	0.1 (0.01–0.36)	−0.5 (−0.6–0.39)*
45–49 years	0.81 (0.11–2.74)	0.71 (0.09–2.33)	−0.42 (−0.5–0.35)*	0.88 (0.12–2.87)	0.73 (0.09–2.48)	−0.68 (−0.77–0.6)*	0.84 (0.11–2.79)	0.72 (0.09–2.32)	−0.55 (−0.62–0.48)*
50–54 years	2.22 (0.37–7.28)	1.95 (0.31–6.05)	−0.37 (−0.48–0.26)*	2.51 (0.41–7.84)	2.12 (0.33–6.71)	−0.51 (−0.6–0.41)*	2.36 (0.39–7.53)	2.03 (0.32–6.27)	−0.43 (−0.53–0.33)*
55–59 years	5.57 (1.01–16.72)	5.12 (0.94–15.34)	−0.11 (−0.25–0.03)	6.4 (1.17–17.97)	5.91 (1.06–17.38)	−0.01 (−0.17–0.14)	5.96 (1.11–17.19)	5.51 (1.01–16.26)	−0.05 (−0.19–0.09)
60–64 years	12.21 (2.58–34.78)	12.03 (2.44–36.82)	0.19 (0.05–0.33)*	14.52 (3.03–42.1)	14.51 (3.01–42.33)	0.29 (0.15–0.42)*	13.33 (2.93–39.45)	13.26 (2.72–40.37)	0.25 (0.12–0.39)*
65–69 years	24.56 (5.35–72.57)	24.41 (5.23–73.23)	0.24 (0.11–0.38)*	30.21 (6.48–89.18)	29.93 (6.43–85.55)	0.26 (0.14–0.38)*	27.43 (6.06–81.42)	27.22 (5.96–80.84)	0.25 (0.13–0.38)*
70–74 years	48.8 (10.7–144.28)	49.35 (10.88–140.16)	0.27 (0.14–0.4)*	62.66 (13.6–177.5)	61.76 (13.74–170.1)	0.2 (0.1–0.29)*	56.23 (12.39–164.79)	55.71 (12.51–160.23)	0.2 (0.1–0.31)*
75–79 years	107.26 (23.73–327.9)	110.28 (24.38–333.83)	0.33 (0.2–0.47)*	146.3 (32.03–423.93)	141.99 (32.6–407.45)	0.14 (0.04–0.25)*	129.5 (28.39–378.68)	126.97 (28.86–358.84)	0.17 (0.06–0.28)*
80–84 years	336.86 (75.54–993.17)	351.28 (78.21–1054)	0.33 (0.21–0.45)*	471.38 (105.65–1379.28)	451.76 (105.36–1245.61)	0.01 (–0.08–0.09)	419.48 (93.81–1253.74)	407.63 (93.39–1133.43)	0.06 (–0.03–0.16)
85–89 years	780.42 (175.7–2264.23)	826.57 (188.84–2375.66)	0.37 (0.27–0.47)*	1062.62 (248.35–2937.88)	1015.43 (238.27–2611.08)	−0.1 (−0.15–0.04)*	974.58 (227.25–2679.16)	949.31 (220.72–2514.2)	0.01 (−0.06–0.08)
Age-standardized rate	19.07 (4.35–53.18)	20.25 (4.8–57.38)	0.42 (0.31–0.53)*	25.42 (5.97–69.18)	24.92 (5.98–64.47)	0.08 (0–0.15)	23.41 (5.4–64.04)	23.32 (5.66–61.31)	0.15 (0.07–0.23)*

*^∗^Changes that are statistically significant.*

### Joinpoint Regression Analysis

The AAPCs in the sex-specific incidence and mortality rates for dementia by age group from 1990 to 2019 are presented in Table 1. From 1990 to 2019, the age-standardized incidence and mortality rates for dementia in China increased by 0.33% (95% CI, 0.27–0.40%) and 0.15% (95% CI, 0.07–0.23%), respectively. The most significant AAPC increase in the incidence rate was observed in the group aged 55–59 years among both males (0.93%; 95% CI, 0.87–0.99%) and females (0.80%; 95% CI, 0.73–0.87%). Moreover, the increments in the AAPCs in the age-standardized incidence [0.49% (95% CI, 0.43–0.55%) vs. 0.31% (95% CI, 0.24–0.38%)] and mortality [0.42% (95% CI, 0.31–0.53%) vs. 0.08% (0.00–0.15%)] rates for dementia in China were higher in males than in females from 1990 to 2019. The AAPCs in the age-specific mortality rates increased with increasing age groups among males, while the highest significant AAPC increase (0.29%; 95% CI, 0.15–0.42%) among females was seen in the group aged 60–64 years (Table 1).

**FIGURE 2:**
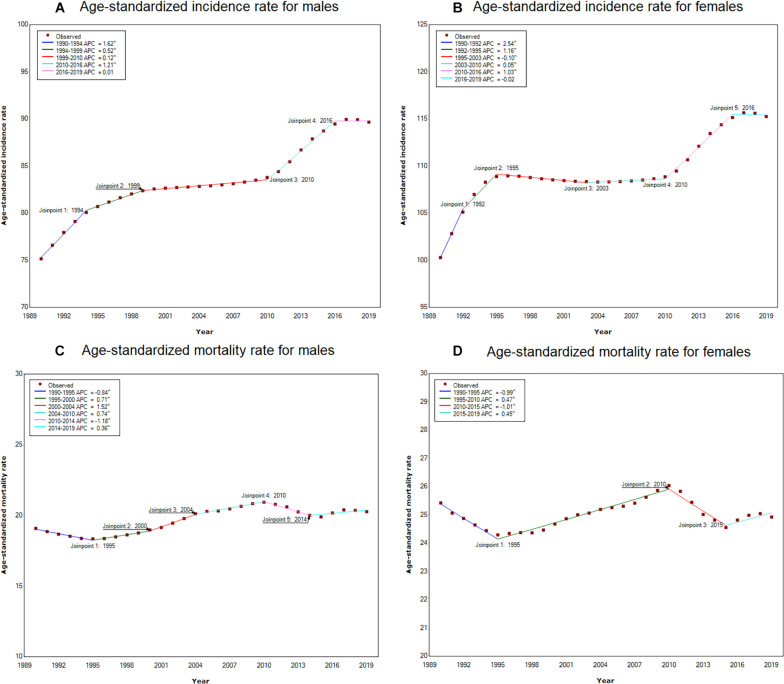
Joinpoint regression analysis of the sex-specific age-standardized incidence and mortality rates for Alzheimer’s disease and other forms of dementia in China from 1990 to 2019. **(A)** Age-standardized incidence rate for males; **(B)** Age-standardized incidence rate for females; **(C)** Age-standardized mortality rate for males; **(D)** Age-standardized mortality rate for females.

Joinpoint regression analysis of the sex-specific, age-standardized incidence and mortality rates for dementia in China from 1990 to 2019 are shown in Figure 2. Generally, the age-standardized incidence rates substantially increased from 2010 to 2016 and then maintained a flat trend during the period from 2016 to 2019 among both males and females. The age-standardized mortality rate significantly decreased and then subsequently increased from 2010 to 2019 in both males and females.

### Age–Period–Cohort Analysis With the Intrinsic Estimator Method

**FIGURE 3:**
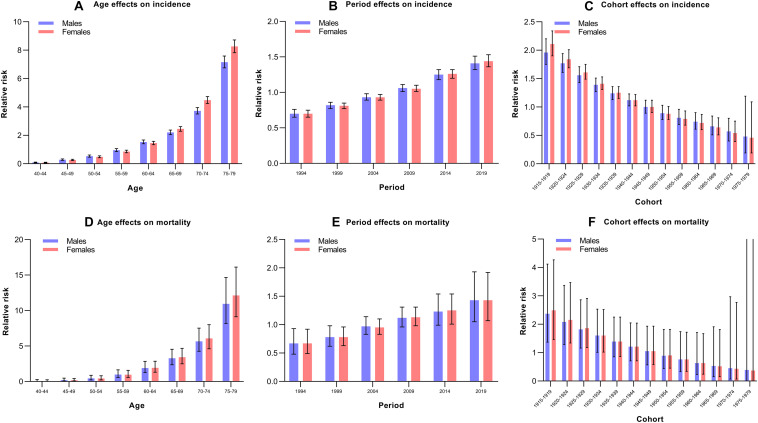
Relative risks of the incidence of and mortality due to Alzheimer’s disease and other forms of dementia in China from 1990 to 2019 due to age, period, and cohort effects. **(A)** Age effects on incidence; **(B)** Period effects on incidence; **(C)** Cohort effects on incidence; **(D)** Age effects on mortality; **(E)** Period effects on mortality; **(F)** Cohort effects on mortality.

The estimated RRs for incident dementia and dementia-associated mortality due to the age, period, and cohort effects are shown in Table 2 and Figure 3. When the period and cohort effects were controlled for, significant positive RRs for incident dementia and dementia-associated mortality were identified in those aged 60 years and older among both males and females. With regard to the period effect, we observed increasing trends in the risk of developing dementia over time among males and females, especially after 2009 (Figure 3). The RRs for mortality associated with period effects showed significant increases in 2019 (RR, 1.42; 95% CI, 1.05–1.93) in males and significantly higher RRs, at 1.25 (95% CI, 1.01–1.54) and 1.43 (95% CI, 1.07–1.92) in 2014 and 2019 for females, respectively (Table 2). Regarding the cohort effect, the RRs for incident dementia and dementia-associated mortality continuously decreased in later birth cohorts in both males and females (Figure 3).

**TABLE 2:** Sex-specific relative risks of the incidence of and mortality due to Alzheimer’s disease and other forms of dementia in China due to age, period, and cohort effects.

Factor	Incidence in males		Incidence in females		Mortality in males		Mortality in females	
	RR (95% CI)	*P*-value	RR (95% CI)	*P*-value	RR (95% CI)	*P*-value	RR (95% CI)	*P*-value
Age								
40–44	0.08 (0.06–0.11)	<0.001	0.07 (0.05–0.10)	<0.001	0.03 (0.00–0.27)	0.002	0.03 (0.00–0.24)	0.001
45–49	0.28 (0.24–0.34)	<0.001	0.26 (0.22–0.31)	<0.001	0.19 (0.08–0.47)	<0.001	0.18 (0.07–0.42)	<0.001
50–54	0.53 (0.46–0.61)	<0.001	0.48 (0.42–0.55)	<0.001	0.44 (0.22–0.87)	0.019	0.42 (0.22–0.81)	0.01
55–59	0.95 (0.85–1.06)	0.398	0.85 (0.77–0.95)	0.003	0.97 (0.58–1.64)	0.915	0.96 (0.58–1.58)	0.857
60–64	1.53 (1.40–1.67)	<0.001	1.45 (1.34–1.58)	<0.001	1.89 (1.26–2.84)	0.002	1.93 (1.30–2.85)	0.001
65–69	2.20 (2.05–2.37)	<0.001	2.45 (2.29–2.61)	<0.001	3.27 (2.36–4.54)	<0.001	3.42 (2.49–4.68)	<0.001
70–74	3.72 (3.50–3.95)	<0.001	4.48 (4.24–4.73)	<0.001	5.65 (4.25–7.52)	<0.001	6.07 (4.60–8.00)	<0.001
75–79	7.15 (6.75–7.59)	<0.001	8.26 (7.82–8.72)	<0.001	10.94 (8.17–14.65)	<0.001	12.13 (9.12–16.13)	<0.001
Period								
1994	0.70 (0.65–0.76)	<0.001	0.70 (0.65–0.75)	<0.001	0.67 (0.48–0.93)	0.017	0.67 (0.49–0.92)	0.012
1999	0.82 (0.77–0.86)	<0.001	0.81 (0.77–0.85)	<0.001	0.78 (0.62–0.98)	0.03	0.78 (0.63–0.96)	0.02
2004	0.93 (0.89–0.98)	0.006	0.93 (0.89–0.97)	<0.001	0.97 (0.83–1.14)	0.714	0.95 (0.83–1.10)	0.498
2009	1.06 (1.01–1.11)	0.027	1.05 (1.01–1.10)	0.019	1.12 (0.96–1.31)	0.158	1.13 (0.98–1.31)	0.101
2014	1.25 (1.18–1.32)	<0.001	1.26 (1.20–1.32)	<0.001	1.23 (0.99–1.54)	0.067	1.25 (1.01–1.54)	0.039
2019	1.41 (1.32–1.51)	<0.001	1.44 (1.36–1.53)	<0.001	1.43 (1.05–1.93)	0.021	1.43 (1.07–1.92)	0.015
Cohort								
1915–1919	1.96 (1.75–2.20)	<0.001	2.11 (1.90–2.34)	<0.001	2.37 (1.37–4.12)	0.002	2.49 (1.46–4.27)	0.001
1920–1924	1.77 (1.61–1.94)	<0.001	1.84 (1.69–2.01)	<0.001	2.08 (1.28–3.37)	0.003	2.15 (1.34–3.47)	0.002
1925–1929	1.56 (1.43–1.71)	<0.001	1.61 (1.49–1.75)	<0.001	1.82 (1.16–2.86)	0.01	1.86 (1.18–2.91)	0.007
1930–1934	1.39 (1.27–1.51)	<0.001	1.41 (1.31–1.53)	<0.001	1.60 (1.01–2.53)	0.043	1.60 (1.02–2.52)	0.042
1935–1939	1.24 (1.13–1.36)	<0.001	1.25 (1.15–1.36)	<0.001	1.39 (0.85–2.25)	0.186	1.39 (0.86–2.25)	0.183
1940–1944	1.12 (1.02–1.23)	0.023	1.12 (1.02–1.22)	0.013	1.21 (0.71–2.05)	0.488	1.21 (0.71–2.04)	0.483
1945–1949	1.00 (0.89–1.120)	0.998	1.00 (0.90–1.12)	0.962	1.05 (0.57–1.93)	0.88	1.05 (0.58–1.93)	0.865
1950–1954	0.89 (0.78–1.03)	0.115	0.88 (0.78–1.01)	0.061	0.89 (0.44–1.82)	0.754	0.90 (0.45–1.82)	0.776
1955–1959	0.81 (0.69–0.96)	0.013	0.79 (0.68–0.93)	0.004	0.76 (0.33–1.74)	0.508	0.76 (0.34–1.72)	0.515
1960–1964	0.74 (0.61–0.90)	0.003	0.72 (0.60–0.87)	0.001	0.63 (0.23–1.71)	0.362	0.63 (0.24–1.67)	0.35
1965–1969	0.66 (0.51–0.84)	0.001	0.64 (0.51–0.81)	<0.001	0.53 (0.15–1.91)	0.332	0.52 (0.15–1.81)	0.302
1970–1974	0.57 (0.40–0.80)	0.001	0.54 (0.39–0.75)	<0.001	0.45 (0.07–2.97)	0.407	0.43 (0.07–2.77)	0.376
1975–1979	0.48 (0.19–1.19)	0.111	0.46 (0.19–1.09)	0.077	0.39 (0.00–141.49)	0.756	0.37 (0.00–135.78)	0.739
Deviance	0.61		0.54		0.03		0.07	
AIC	7.3		7.5		4.78		4.93	
BIC	−92.29		−92.37		−92.88		−92.84	

*RR denotes the relative risk of the incidence and mortality in a particular age, period, or birth cohort relative to the average level of all ages, periods, or birth cohorts combined. RR, relative risk; CI, confidence interval; AIC, Akaike Information Criterion; BIC, Bayesian Information Criterion.*

## Discussion

To our knowledge, this is the first study to explore the long-term trends in the incidence of and mortality due to Alzheimer’s disease and other forms of dementia in China from 1990 to 2019 using the age-period-cohort framework based on data from the GBD 2019 study. This study found that the age-standardized incidence and mortality rates for Alzheimer’s disease and other forms of dementia significantly increased from 1990 to 2019 in China, with AAPCs of 0.33 and 0.15%, respectively. The minor increase in the incidence rate is similar to that reported in a previous study in China that investigated the period from1990 to 2010 (Chan et al., 2013). However, these findings were inconsistent with incidence trend studies in Western European countries, the United States and Nigeria (Schrijvers et al., 2012; Gao et al., 2016; Grasset et al., 2016; Matthews et al., 2016; Satizabal et al., 2016; Wu et al., 2017). These population-based studies have reported stable or declining incidence rates and evidence of both inconsistent and similar changes in men and women within and across countries (Schrijvers et al., 2012; Gao et al., 2016; Grasset et al., 2016; Matthews et al., 2016; Satizabal et al., 2016; Wu et al., 2017). It is difficult to provide a unifying explanation for these different results; however, we assume that the differences might be explained by variations in economic development; political, social and cultural backgrounds; and the pace of change over the past few decades among these countries (Schrijvers et al., 2012; Gao et al., 2016; Grasset et al., 2016; Matthews et al., 2016; Satizabal et al., 2016; Wu et al., 2017). First, given the association between smoking and the risk of dementia, the increasing prevalence of smoking in the Chinese population may have contributed to a different outcome than in Western countries (Wang Y.Q. et al., 2019). A second explanation, compared with the United States, lower average years of education among the elderly in China might explain some of the increase in incidence and mortality (Wu et al., 2017). Finally, population-level investments in infrastructure, education, health services and social welfare contribute to substantial improvements in many aspects of physical, mental and cognitive health from early life and may therefore reduce the risk of dementia in later life (Wu et al., 2017). However, China’s economic development and infrastructure construction started late, and compared with Western developed countries, individuals in the early period were exposed to more risk factors. We observed that the age-standardized mortality rate significantly increased in males but remained stable in females. These findings are inconsistent with those of a previous study that reported that mortality due to Alzheimer’s disease and other forms of dementia decreased from 2009 to 2015 based on data from the National Mortality Surveillance System (Bo et al., 2019). These differences in trends between the present study and previous studies may be attributable to different sources and degrees of completeness of the mortality data in China. Furthermore, the differences may be related to the duration of the observation period.

In this study, the age-standardized incidence was higher among females than among males in 2019. This was consistent with previous studies (Chan et al., 2013). The age-standardized global prevalence in females was 1.17 times the age-standardized prevalence in males in 2016, which indicates that more women are affected by dementia than men worldwide (GBD 2016 Disease and Injury Incidence and Prevalence Collaborators, 2017). Sex differences have also been found in some other prevalence and incidence trend studies (Wu et al., 2017). Three European studies reported decreasing trends in prevalence in men but mixed results in women (Lobo et al., 2007; Matthews et al., 2013; Wimo et al., 2016). With regard to incidence studies, mixed findings in men and women have been reported in the United States and the Netherlands (no difference), the United Kingdom (declining trend in men), and France and the Framingham Heart Study (greater or earlier decrease in women) (Schrijvers et al., 2012; Gao et al., 2016; Grasset et al., 2016; Matthews et al., 2016; Satizabal et al., 2016). The age-standardized mortality rates in women were also higher than those in men, which is in line with a higher prevalence in women than in men, indicating that the female predominance is not simply due to the longer lifespan of women. Globally, more women than men died from dementia in 2016 (GBD 2016 Disease and Injury Incidence and Prevalence Collaborators, 2017). The differences may be due to the physiological differences between men and women (Ballard et al., 2011). These findings have important policy implications because women in China have a considerably longer life expectancy than men and constitute up to 75% of the population of people aged 85 years and older (Chan et al., 2013). However, we also found that males had higher AAPCs in the incidence of and mortality due to Alzheimer’s disease and other forms of dementia than females in the current study. This may reveal a potential increasing burden of dementia in men. Therefore, given the higher prevalence of smoking among men than among women, improvements in the prevention and treatment of cardiovascular diseases may have a greater impact on men’s health and life expectancy than women’s, and this major change in a risk factor for brain impairment and dementia is worthy of further study (Wu et al., 2017).

Generally, the age effect explained why the incidence and mortality increased with increasing age among males and females. These findings were supported by a previous review (Ballard et al., 2011). The current study indicated that the disease burden of dementia is increasing due to the increasing number of people living with Alzheimer’s disease and other forms of dementia, and the primary reason is the aging of the population. Considering the rapid aging of the Chinese population, the disease burden caused by Alzheimer’s disease and other forms of dementia will increase rapidly in the coming decades (Keogh-Brown et al., 2015). As reported by the World Alzheimer Report in 2010, the societal costs of Alzheimer’s disease and other forms of dementia are almost as high as those of cancer, heart disease and stroke (World Alzheimer Report, 2010). Due to the increasing aging of the global population, social costs may soar in the future.

The period effect is usually due to a series of complex historical events and environmental factors. We observed increasing trends in the risk of developing dementia over time among males and females, especially after 2009. This may be due to environmental changes and a more rapid pace of life. Lifestyle factors have changed substantially in China, including the impairment of sleep hygiene and the adoption of unhealthy eating habits (Bo et al., 2019). These changes increase the risk of suffering from various chronic diseases, such as diabetes, stroke and hypertension, which are strongly correlated with the onset of Alzheimer’s disease and other forms of dementia (Wang et al., 2017, 2018; Li et al., 2020). Recent studies have reported that the prevalence of diabetes, obesity, and hypertension has increased over the last decade (Wang et al., 2017, 2018; Li et al., 2020).

The cohort effect on the incidence of and mortality due to dementia revealed continuously decreasing trends in later birth cohorts in both males and females. The possible reason was that the later birth cohorts received better education and had a greater awareness of health and disease prevention than earlier birth cohorts (Cohen and Syme, 2013). However, the level of education may not adequately reflect the cognitive abilities of a person when comparing different birth cohorts. The older cohort probably had fewer possibilities to continue education after primary school, regardless of their intellectual abilities. In addition, the implementation of preventive treatments and reduction in vascular risk factors at the population level may have influenced the decreasing incidence of stroke in China (Liu et al., 2020). In a previous study, vascular risk factors were more prevalent in the later birth cohort than in the earlier birth cohort (Schrijvers et al., 2012). This was, however, paralleled by an increase in the use of antithrombotic and lipid-lowering drugs. The use of statins has been previously reported to be associated with a lower risk of dementia, and both antithrombotic and lipid-lowering drugs are preventive treatments for cerebrovascular disease, thereby providing a potential explanation for the decreasing trend across cohorts (Green et al., 2006). However, the reason for the trends in incidence and mortality across cohorts must be interpreted with caution. Improved strategies are needed for earlier birth cohorts who face a higher risk.

Our current study had certain limitations that might have affected the accuracy of our results. First, bias and gaps could not be avoided in the modeling process related to the dementia burden, as described previously (GBD 2019 Diseases and Injuries Collaborators, 2020; GBD 2019 Risk Factors Collaborators, 2020). Additionally, the data sources, coding practices with regard to the cause of disease and definitions of dementia were heterogeneous. Second, the cases were not divided into dementia subtypes; therefore, the age-period-cohort analysis considered a community as the observed and analyzed unit, which might have resulted in ecological fallacies. Therefore, our results in the present study on the epidemiology of Alzheimer’s disease and other forms of dementia should be interpreted with caution.

## Conclusion

In conclusion, the age-standardized incidence rate of Alzheimer’s disease and other forms of dementia increased in China among both males and females during the 1990–2019 period, with the highest AAPC in the group aged 55–59 years. The age-standardized mortality rate significantly increased in males. Notably, males had higher AAPCs in the incidence of and mortality due to Alzheimer’s disease and other forms of dementia than females. In addition, older age is associated with the highest risk of developing dementia. The period effect indicated that the risk of developing dementia continuously increased with time. An enhanced understanding of risk profiles and onset patterns associated with Alzheimer’s disease and other forms of dementia could facilitate the early identification of individuals who are at risk of developing dementia, thereby supporting the timely initiation of interventions that could effectively reduce the dementia burden.

## Data Availability Statement

The datasets presented in this study can be found in online repositories. The names of the repository/repositories and accession number(s) can be found below: http://ghdx.healthdata.org/gbd-results-tool.

## Author Contributions

YG and XL conceived and designed the study. XL supervised the study. YG did the statistical analysis and drafted the manuscript. Both authors contributed to acquisition, analysis, and interpretation of data, revised the report, and approved the final version before submission.

## Conflict of Interest

The authors declare that the research was conducted in the absence of any commercial or financial relationships that could be construed as a potential conflict of interest.

## Publisher’s Note

All claims expressed in this article are solely those of the authors and do not necessarily represent those of their affiliated organizations, or those of the publisher, the editors and the reviewers. Any product that may be evaluated in this article, or claim that may be made by its manufacturer, is not guaranteed or endorsed by the publisher.
