# Supplementary material for: Secular Trends in the Incidence of and Mortality Due to Alzheimer’s Disease and Other Forms of Dementia in China From 1990 to 2019: An Age-Period-Cohort Study and Joinpoint Analysis
Source: Front Aging Neurosci. 2021 Sep 3;13:709156. doi: 10.3389/fnagi.2021.709156 (PMC8446521; doi:10.3389/fnagi.2021.709156)
Supplement: Supplementary file 1 [file Data_Sheet_1.docx]

**Supplement. Overview for Global Burden of Disease 2019**

The Global Burden of Disease (GBD) is an approach to global descriptive epidemiology [1]. It is a systematic, scientific effort to quantify the comparative magnitude of health loss due to diseases, injuries, and risk factors by age, sex, and geographies for specific points in time. IHME serves as the coordinating center for the GBD and affiliated projects. Published in The Lancet in October 2020, GBD 2019 provides for the first time an independent estimation of population, for each of 204 countries and territories and the globe, using a standardized, replicable approach, as well as a comprehensive update on fertility and migration [1]. GBD 2019 incorporates major data additions and improvements, and methodological refinements. Mortality and life expectancy estimates have expanded to a total of 990 locations at the most detailed level, and new causes have been added to the fatal and non-fatal cause lists, for a total of 369 diseases and injuries (http://www.healthdata.org/gbd/about/protocol). GBD 2019 estimated each epidemiological quantity of interest—incidence, prevalence, mortality, years lived with disability (YLDs), years of life lost (YLLs), and disability-adjusted life-years (DALYs)—for 23 age groups; males, females, and both sexes combined; and 204 countries and territories that were grouped into 21 regions and seven super-regions. GBD 2019 location hierarchy now includes all WHO member states. The GBD diseases and injuries analytical framework generated estimates for every year from 1990 to 2019. Diseases and injuries were organised into a levelled cause hierarchy from the three broadest causes of death and disability at Level 1 to the most specific causes at Level 4. Within the three Level 1 causes—communicable, maternal, neonatal, and nutritional diseases; non-com mu-nicable diseases; and injuries—there are 22 Level 2 causes, 174 Level 3 causes, and 301 Level 4 causes (including 131 Level 3 causes that are not further disaggregated at Level 4). 364 total causes are non-fatal and 286 are fatal [1].

**Definition of indicator**

Dementia is a progressive, degenerative, and chronic neurological disorder typified by memory impairment and other neurological dysfunctions. For the purposes of GBD 2019, they use the Diagnostic and Statistical Manual of Mental Disorders III, IV or V, or ICD case definitions as the reference. The DSM-IV definition is:

• Multiple cognitive deficits manifested by both memory impairment and one of the following: aphasia, apraxia, agnosia, disturbance in executive functioning

• Must cause significant impairment in occupational functioning and represent a significant decline.

• Course is characterized by gradual onset and continuing cognitive decline

• Cognitive deficits are not due to other psychiatric conditions

• Deficits do not occur exclusively during the course of a delirium

A wide array of diagnostic and screening instruments exists, including Clinical Dementia Rating scale (CDR), Mini Mental State Examination (MMSE), and the Geriatric Mental State (GMS). For severity rating purposes we use the CDR as the reference. The relevant ICD-10 codes for dementia are F00, F01, F02, F03, G30, and G31. The ICD-9 codes are 290, 291.2, 291.8, 294 and 331.

**Data sources**

The GBD estimation process is based on identifying multiple relevant data sources for each disease or injury including censuses, household surveys, civil registration and vital statistics, disease registries, health service use, air pollution monitors, satellite imaging, disease notifications, and other sources. Each of these types of data are identified from systematic review of published studies, searches of government and international organisation websites, published reports, primary data sources such as the Demographic and Health Surveys, and contributions of datasets by GBD collaborators. 86249 sources were used in this analysis, including 19354 sources reporting deaths, 31499 reporting incidence, 1973 reporting prevalence, and 26631 reporting other metrics. Each newly identified and obtained data source is given a unique identifier by a team of librarians and included in the Global Health Data Exchange (GHDx). The GHDx makes publicly available the metadata for each source included in GBD as well as the data, where allowed by the data provider. Additional metadata for each source are available in the online GBD citation tool, http://ghdx.healthdata.org/gbd-results-tool.

**Modelling**

For most diseases and injuries, processed data are modelled using standardised tools to generate estimates of each quantity of interest by age, sex, location, and year [1]. There are three main standardised tools: Cause of Death Ensemble model (CODEm), spatiotemporal Gaussian process regression (ST-GPR), and DisMod-MR. Previous publications provide more details on these general GBD methods [2-4]. Briefly, CODEm is a highly systematised tool to analyse cause of death data using an ensemble of different modelling methods for rates or cause fractions with varying choices of covariates that perform best with out-of-sample predictive validity testing. DisMod-MR is a Bayesian meta-regression tool that allows evaluation of all available data on incidence, prevalence, remission, and mortality for a disease, enforcing consistency between epidemiological parameters. ST-GPR is a set of regression methods that borrow strength between locations and over time for single metrics of interest, such as risk factor exposure or mortality rates [1].

**Reference**

1. GBD 2019 Diseases and Injuries Collaborators. Global burden of 369 diseases and injuries in 204 countries and territories, 1990-2019: a systematic analysis for the Global Burden of Disease Study 2019. Lancet. 2020 Oct 17;396(10258):1204-1222. doi: 10.1016/S0140-6736(20)30925-9.
2. GBD 2017 Disease and Injury Incidence and Prevalence Collaborators. Global, regional, and national incidence, prevalence, and years lived with disability for 354 diseases and injuries for 195 countries and territories, 1990-2017: a systematic analysis for the Global Burden of Disease Study 2017. Lancet. 2018 Nov 10;392(10159):1789-1858. doi: 10.1016/S0140-6736(18)32279-7.
3. GBD 2017 Causes of Death Collaborators. Global, regional, and national age-sex-specific mortality for 282 causes of death in 195 countries and territories, 1980-2017: a systematic analysis for the Global Burden of Disease Study 2017. Lancet. 2018 Nov 10;392(10159):1736-1788. doi: 10.1016/S0140-6736(18)32203-7.
4. GBD 2017 Diet Collaborators. Health effects of dietary risks in 195 countries, 1990-2017: a systematic analysis for the Global Burden of Disease Study 2017. Lancet. 2019 May 11;393(10184):1958-1972. doi: 10.1016/S0140-6736(19)30041-8.
